# Nanomechanical mechanisms of Lyme disease spirochete motility enhancement in extracellular matrix

**DOI:** 10.1038/s42003-021-01783-1

**Published:** 2021-03-01

**Authors:** Martin Strnad, Yoo Jin Oh, Marie Vancová, Lisa Hain, Jemiina Salo, Libor Grubhoffer, Jana Nebesářová, Jukka Hytönen, Peter Hinterdorfer, Ryan O. M. Rego

**Affiliations:** 1 Biology Centre ASCR, v.v.i., Ceske Budejovice, Czech Republic; 2 Faculty of Science, University of South Bohemia, Ceske Budejovice, Czech Republic; 3 Institute of Biophysics, Johannes Kepler University Linz, Linz, Austria; 4 Institute of Biomedicine, University of Turku, Turku, Finland; 5 Laboratory Division, Clinical Microbiology, Turku University Hospital, Turku, Finland

**Keywords:** Pathogens, Cellular motility

## Abstract

As opposed to pathogens passively circulating in the body fluids of their host, pathogenic species within the Spirochetes phylum are able to actively coordinate their movement in the host to cause systemic infections. Based on the unique morphology and high motility of spirochetes, we hypothesized that their surface adhesive molecules might be suitably adapted to aid in their dissemination strategies. Designing a system that mimics natural environmental signals, which many spirochetes face during their infectious cycle, we observed that a subset of their surface proteins, particularly Decorin binding protein (Dbp) A/B, can strongly enhance the motility of spirochetes in the extracellular matrix of the host. Using single-molecule force spectroscopy, we disentangled the mechanistic details of DbpA/B and decorin/laminin interactions. Our results show that spirochetes are able to leverage a wide variety of adhesion strategies through force-tuning transient molecular binding to extracellular matrix components, which concertedly enhance spirochetal dissemination through the host.

## Abstract (web summary)

Martin Strnad, Yoo Jin Oh, and colleagues use single-molecule force spectroscopy and an extracellular matrix (ECM) analog that mimics natural tick feeding to show that the surface proteins DbpA/B can enhance spirochete motility in the ECM of the host. These results show that spirochetes can tune their transient molecular binding to ECM components to enhance spirochetal dissemination through the host.

## Introduction

Bacterial adhesins are cell-surface components that facilitate adhesion to other cells or surfaces. The conventional viewpoint on adhesins is that they determine bacterial attachment and enable them to resist physical removal by shear stress caused by hydrodynamic shear forces^1^. To maximize their contact with the environment, adhesins are often present on outward hairlike structures such as pili and fimbriae. Spirochetes do not possess such external structures. Additionally, spirochetes differ from most other motile pathogenic bacteria in that the spirochetes miss external appendages that are commonly required for bacterial motility. The unique corkscrew rotational movement is generated by periplasmic flagella hidden beneath the outer membrane, which allows them to swim in highly viscous, gel-like media that slow down or stop most bacteria with external flagella^2^.

A typical representative of pathogenic spirochetes, *Borrelia burgdorferi*, expresses several adhesins that enable contact with its vertebrate hosts^3^. Decorin-binding proteins (DbpA and DbpB) and fibronectin-binding proteins BBK32 and RevA belong to the most recognized and functionally better characterized adhesins^4–6^. DbpA and DbpB mutants show significant attenuation in mice, particularly early in infection^7^. Disruption of *dbpA* and *dbpB* decrease recovery of spirochetes from tissues distant to the inoculation site^8^. Similarly, *revA*-deficient spirochetes disseminate significantly less to distal organs^9^. BBK32 mutant exhibits a decrease in mice colonization and a delay in dissemination when compared to the parental strain^10^.

The common denominator, for all four adhesins in the context of infection, is delayed dissemination and colonization, particularly of distal tissues. Until now, the reason for this remains largely obscure as it was differently attributed to the effects of acquired immunity^7^, innate immunity^8^, or the inability to adhere properly to host ECM components^8,10^. Surprisingly, relating the dynamic-binding properties of adhesins to borrelial motility or propagation within the host has never been attempted, possibly due to the absence of a quantitative assay that would allow to reliably mimic the movement of the spirochete through the host tissues. The goal here was to pursue the assumption that adhesins not only provide stationary attachments alone but also temporarily enhance the movement of the spirochetes.

## Results

### Adhesin expression does not enhance motility in standard in vitro assays

**Fig. 1:**
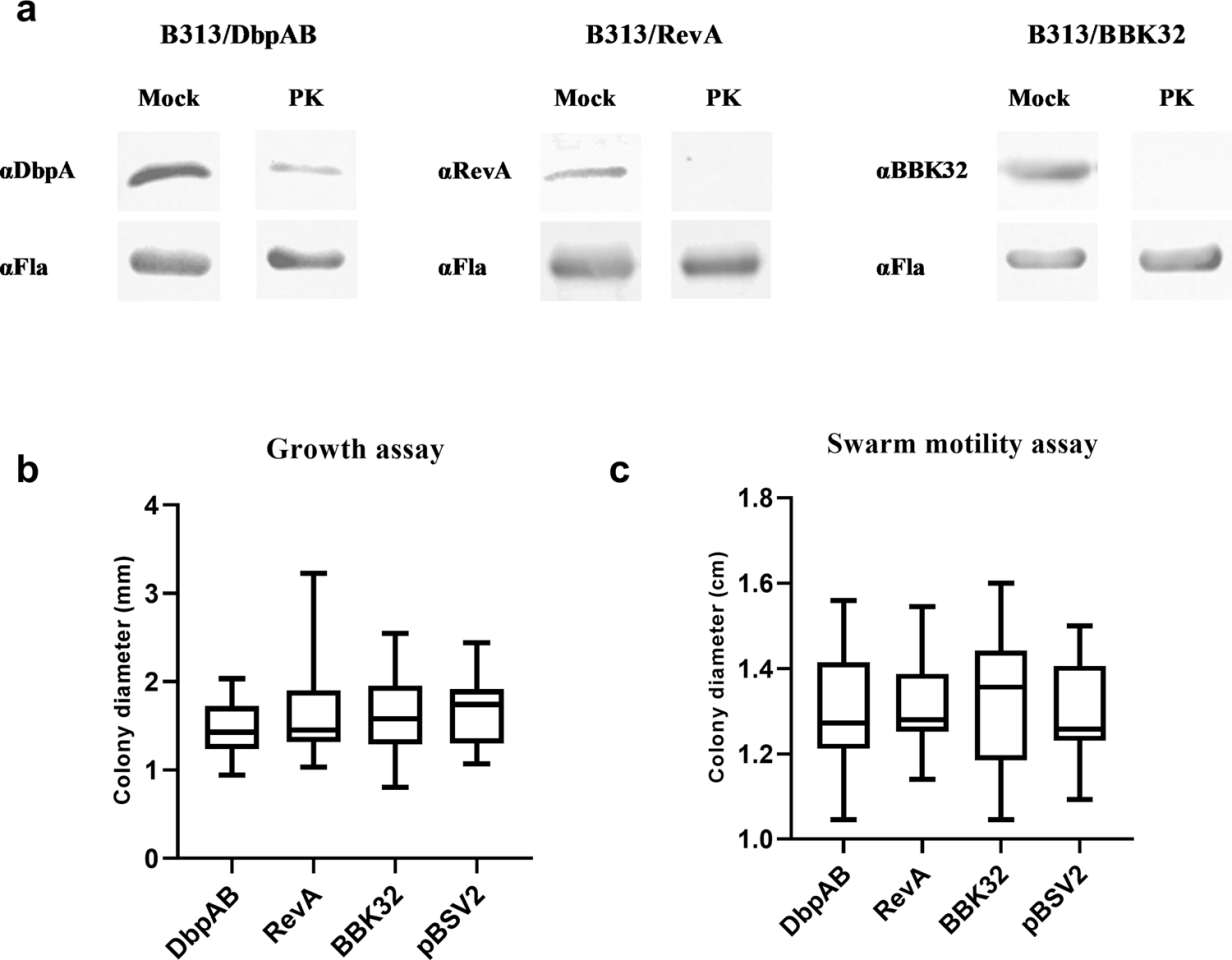
Expression of adhesins does not enhance undirected motility in standard in vitro conditions. **a** To determine if DbpAB, BBK32, and RevA proteins are present in borrelial lysates and are surface exposed, a proteinase K (PK) assay was employed. PK-treated and -untreated lysates of recombinant *B. burgdorferi* B313 strains (B313/DbpAB, B313/RevA, B313/BBK32) expressing DbpA and DbpB, RevA, and BBK32 were separated by SDS-PAGE and immunoblotted with the indicated antibodies (α, anti). Bands corresponding to specific proteins are absent or faint from PK treated samples. The intensity of subsurface flagellar protein bands are identical between mock and PK treated cells, indicating surface localization of the adhesins. **b**, **c** The effect of DbpAB, RevA, and BBK32 on the undirected movement of spirochetes was studied using growth and swarm motility assays. Empty shuttle vector pBSV2 was used as the control. Results are expressed as arithmetic mean and were compared by one-way ANOVA. Error bars, standard deviation. In the growth assay, 30 colony diameters were measured for each mutant strain. In the swarm motility assay, 12 diameters for each strain were measured. Differences were not statistically significant (*P* > 0.05).

We set out to study the potential influence of selected adhesins on borrelial motility by generating three *Borrelia* adhesin expression mutants. The *revA* and *bbk32* genes were inserted into the shuttle vector pBSV2 and transformed into adhesin-less *B. burgdorferi* B313 to generate B313/RevA and B313/BBK32. B313/DbpAB has been tested already elsewhere^6^. Immunoblotting of bacterial lysates with antiserum raised against recombinant DbpA, RevA, or BBK32 revealed that all adhesin expression mutants produced the respective proteins. The presence of DbpA and DbpB on the surface of B313/DbpAB was shown earlier^6^, and corroborated in this study using a proteinase K (PK) assay. With the same strategy, the surface localization of RevA and BBK32 in B313/RevA and B313/BBK32, respectively, was confirmed (Fig. 1a).

Plate assays are commonly utilized to quantitatively examine bacterial motility on agar plates based on circular turbid zones formed by spirochetes migrating away from the point of cell seeding^11^. To estimate the effect of adhesin expression on borrelial motility, two standard experimental approaches to characterize the undirected movement were performed. Both growth assay (Fig. 1b) and swarm motility assay (Fig. 1c) showed that motility of all adhesin expression mutants (B313/DbpAB, B313/RevA, B313/BBK32) was not significantly altered compared to the parental wild-type cells, and compared to each other. These data show that the adhesins expression does not enhance undirected motility in standard in vitro conditions.

### Spirochetal motility is enhanced in near-natural conditions by DbpA/B

**Fig. 2:**
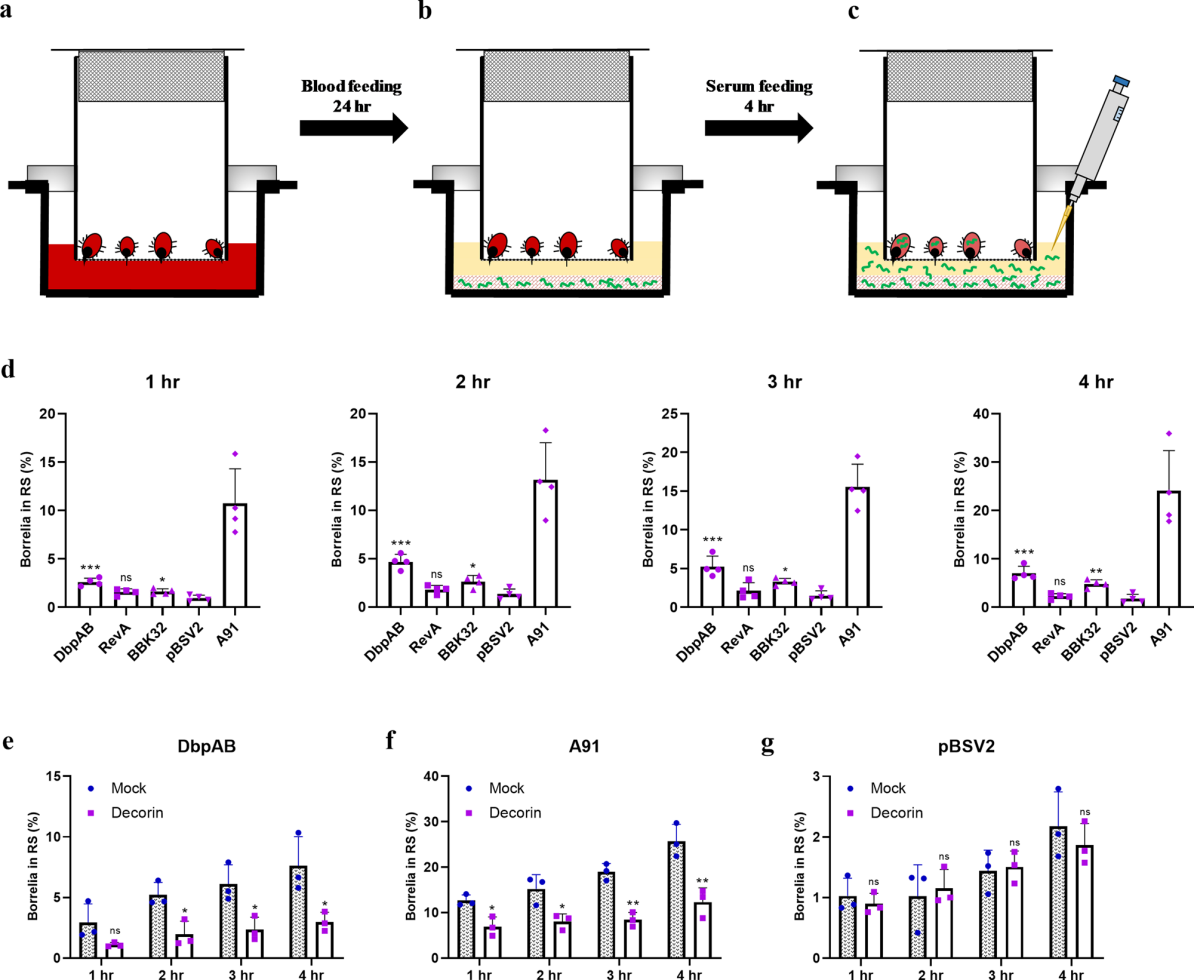
DbpAB expression strongly enhances the number of spirochetes reaching blood serum upon feeding. **a**–**c** Schematic design of the ECM motility assay that imitates the period before *B. burgdorferi* acquisition by ticks. Tick feeding was induced by placing the ticks on fresh rabbit blood for 24 h (**a**). *Borrelia* was embedded in the ECM gel and overlaid with rabbit serum (RS) (**b**). Ticks were allowed to feed and samples of RS were collected in duplicates (2 × 200 μL) at 1-h interval, in total for 4 h (**c**). **d** The number of spirochetes that reached RS was monitored over time using qPCR and the influence of adhesin expression was assessed. The values are given as a percentage of the total number of spirochetes seeded to the ECM gel. The results show that addition of *dbpAB* (and also *bbk32*) into the specific adhesin-free *B. burgdorferi* B313 significantly enhances the motility of the bacterium, but does not fully restore the motility to the level of the infectious strain *B. afzelii* A91. pBSV2—*B. burgdorferi* B313 carrying empty shuttle vector was used as the control. Results are expressed as arithmetic mean and were compared by one-way ANOVA with Tukey *post hoc* test with pBSV2 as a control column. Error bars, standard deviation of four experiments. *P < 0.05; **P < 0.01; ***P < 0.001. The data showed that DbpAB has the most pronounced effect on borrelial motility in ECM. **e**–**g** To determine the effect of DbpAB-ECM interactions on the translational motion of spirochetes, the DbpAB sites were blocked by binding to soluble decorin. The results of the inhibition assays show that motility is significantly hampered in B313/DbpAB (**e**) and control DbpAB-expressing wild-type *B. afzelii* A91 (**f**), marking the importance DbpAB–ECM interactions for borrelial motility. In the control experiment, *B. burgdorferi* B313 with empty shuttle vector pBSV2 was not significantly affected by soluble decorin (**g**). Results are expressed as arithmetic mean and were compared by unpaired Student’s *t* test. Error bars, standard deviation of three experiments. *P < 0.05; **P < 0.01; ns not statistically significant.

The standard methods that are used in studying the specific gene effects on borrelial motility are solely in vitro studies, lacking the vast majority of components and environmental signals which the spirochete faces during its infectious cycle. To bridge the gap between controllable in vitro motility assays and the natural environments that *B. burgdorferi* encounters, we designed a feeding setup mimicking the natural tick feeding on an infected host by using a natural ECM analog (Fig. 2a–c). This system allows us to imitate the migration of *Borrelia* in a host at the time of spirochete acquisition by a tick and reliably assess and quantify the effects of adhesin expression on borrelial motility. Tick feeding was induced by placing *Ixodes ricinus* ticks on fresh rabbit blood for 24 h (Fig. 2a). This time period ensured stable tick feeding as an attachment in membrane-feeding systems is often delayed compared to attachment onto hosts. Next, the spirochetes were embedded in the ECM matrix and overlaid with rabbit serum (RS) (Fig. 2b). As the primary goal of this study was to estimate the effect of adhesins solely on the translational movement of *Borrelia* in the ECM matrix, inactivated RS was used instead of blood during next feeding stages. Blood is known to contain many components with adverse effects on the spirochetes that could reduce their vitality/motility^12^. Moreover, *I. ricinus* is known to feed well on blood serum^13^.

Ticks were allowed to feed and samples of RS were collected in duplicates at 1-h intervals, for a total of 4 h (Fig. 2c). Intriguingly, we observed that the surface presence of two studied borrelial adhesins, DbpAB and BBK32 from *B. afzelii* A91, increase significantly the number of spirochetes in RS and, therefore, the motility of the bacteria, as evidenced by quantitative PCR (Fig. 2d). Spirochete burdens of B313/DbpAB in RS were significantly higher (approximately 3 times) than control group B313/pBSV2 already at 1 hr after spirochete placement, indicating the immediate effect of DbpAB on spirochete motility. Presence of BBK32 also significantly enhanced the motility but the effect was not as strong as of DbpAB. RevA had no significant effect on borrelial motility. To determine whether the enhanced translational motion of spirochetes is caused due to certain specific DbpAB-ECM interactions or just due to the sheer presence of DbpAB, an inhibition experiment was performed. Blocking the availability of DbpAB by soluble decorin resulted in a significant reduction of motility in B313/DbpAB (Fig. 2e) and *B. afzelii* A91 (positive control; Fig. 2f) but not in DbpAB-less B313/pBSV2 (negative control; Fig. 2g). Together, these data show that the enhancement of borrelial motility is caused by certain DbpAB-ECM interactions.

### DbpA/B show stronger interaction with decorin than with laminin in single-molecule bond analysis

**Fig. 3:**
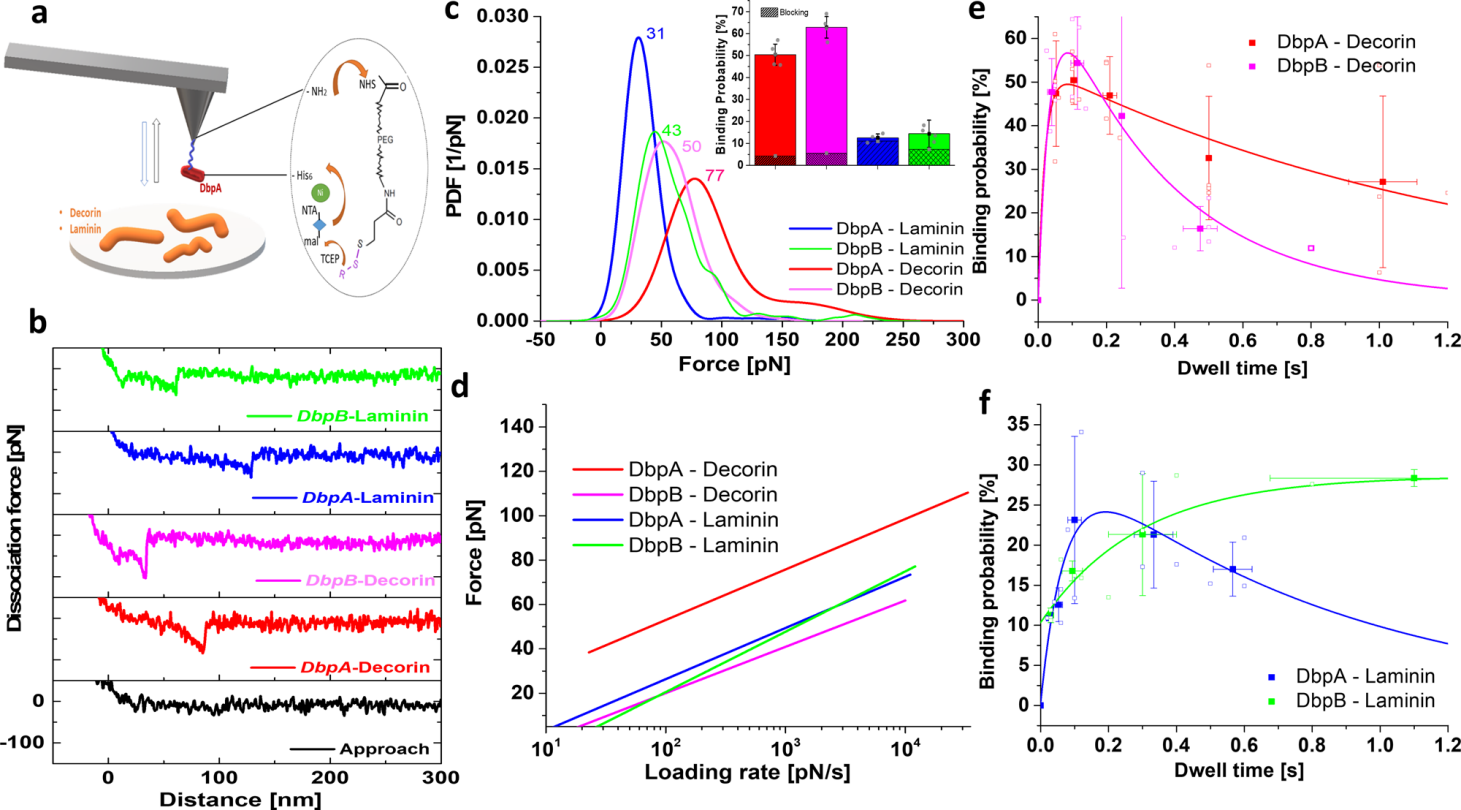
Single molecular Dbps/ECM bond analysis reveals force-tuned dissociation paths. **a** Scheme of the immobilization strategy of decorin binding protein coupled to an amino-functionalized AFM tip end via a heterobifunctional PEG linker, and attachment of extracellular matrix protein onto a silicon substrate. **b** typical force–distance curves obtained by SMFS, from which the dissociation forces (visible as spike at about 50–150 nm distance) of individual Dbps/ECM bonds were measured. Dissociation occurred at different distances, reflecting variable stretching lengths of decorin or laminin. **c** Force distributions depicted as experimental probability density functions (PDFs) were constructed from adding dissociation forces represented by Gaussians of unitary area with widths σ representing the measurement noise (cantilever thermal fluctuation). For each PDF at least 1000 force measurements were recorded at a retraction velocity of 1000 nm/s. PDFs are equivalents to continuos histograms with their maxima being the most probable dissociation forces (see indicated numbers in graph) their uncertainties (widths) reflecting the stochastic nature of the dissociation process. Inset. Binding probabilities (from *n* = 5000 force–distance cycles for each condition, 3–5 different tips), calculated as percentages of force–distance cycles monitoring dissociation forces out of the overall force–distance cycles performed, determined at a tip-surface dwell time of ~0.05 s. After addition of blocking agents (Dbps) into the bath solution, the binding probability dramatically decreased to around 5%, evidencing that binding was of specific nature. **d** dissociation force vs. force loading rate for single Dbps/ECM bonds, i.e., DbpA/decorin, DbpB/decorin, DbpA/laminin, DbpB/laminin. A maximum likelihood approach was used to fit the data and to extract the kinetic off-rate constant (*K*_off_) and the length of the dissociation path (*X*_β_) (see Table 1), using the equation of Bell and Evans^24,25^ (original data and fits are shown in Supplementary Fig. 2), **e**, **f** binding probability assay (BPA). Dbps/ECM bond formation as a function of the dwell time, **e** DbpA/decorin and DbpB/decorin, **f** DbpA/laminin and DbpB/laminin.

Borrelial adhesins are known to bind to multiple ECM ligands^14^. Therefore, we first confirmed that DbpA and DbpB interact and bind components of the ECM gel (Supplementary Fig. 1a). Further, adherence to a number of highly abundant components of the ECM (laminin, fibronectin, collagen, and decorin) was tested using microtiter plate assay, revealing that the Dbps showed efficient binding to decorin and laminin (Supplementary Fig. 1b). To investigate the underlying interaction characteristics of DbpA and DbpB with decorin and laminin as representatives of the ECM components in detail, we utilized the single-molecule force spectroscopy (SMFS) technique^15–18^, based on the wide use of atomic force microscopy (AFM) in microbiology^19–23^. SMFS directly measures dissociation forces by mechanically pulling on molecular interaction bonds. Borrelial surface proteins were conjugated to AFM tips and ECM analogs to surfaces, respectively, via a 6 nm long flexible PEG linker (Fig. 3a) to equip the molecules with sufficient motional freedom for unconstrained specific binding. We performed consecutive force-distance cycles, during which an AFM tip carrying a borrelial surface protein (DbpA, DbpB) was brought into contact with a surface coated with ECM analogs (decorin, laminin) so that a borrelial surface protein/ECM bond was eventually formed. From subsequently retracting the AFM tip from the surface and pulling on the bond with defined speed, the molecular bond was broken at a characteristic measurable dissociation force (Fig. 3b). In force distributions derived from the collection of dissociation forces (Fig. 3c), we found that binding of DbpA was stronger to decorin than to laminin. DbpB showed larger dissociation forces with decorin than with laminin as well. The superior binding capacity of Dbps to decorin was also evidenced from the binding activity: the binding probability values were generally larger for decorin than for laminin binding (Inset, Fig. 3c).

To decipher molecular dynamic and structural features of the Dbps/ECM bonds, we extended our SMFS studies to dynamic force spectroscopy (DFS) experiments and varied the pulling speed. Dissociation forces were measured and individually plotted vs. their force loading rates (equal to pulling velocity times effective spring constant) (Supplementary Fig. 2). In line with Evan’s theory that a single energy barrier is crossed in the thermally activated regime, a linear rise of the dissociation force with respect to a logarithmically increasing loading rate was found (Fig. 3d). Averaging the data fits using the equation of Bell and Evans^24,25^ (Fig. 3d), yielded the kinetic off-rate constant (*K*_off_) extrapolated to zero-force and the length of the force-driven dissociation path (*X*_β_) for Dbps binding to decorin and laminin, respectively (see Table 1). Although more sophisticated models are available for complex interactions, the single energy barrier model^24,25^ fitted well with our data. *X*_β_ was similar for all interactions, indicating comparable dissociation lengths during force-induced bond breakages. In contrast, *K*_off_ values showed pronounced differences. To account for the temporal stabilities of the bonds, we calculated average bond lifetimes, *τ*, directly from the kinetic off-rates, *K*_off_, using the relation $\tau = 1/K_{\mathrm{off}}$ (eq. 1) (Table 1). The lifetime of the DbpB/decorin bond (0.9 s) was slightly larger than that of DbpB/laminin (0.6 s). DbpA/decorin (25.6 s) complexes, however, were strikingly more stable when compared to DbpA/laminin (2.3 s), in line with the ultimately higher dissociation forces required to disrupt this strong bond (Fig. 3c, d).

**Table 1:** Quantification of obtained parameters with dynamic force spectroscopy (DFS) and binding probability assay (BPA) methods.

		DbpA-decorin	DbpB-decorin	DbpA-laminin	DbpB-laminin
*DFS*	*K*_off_ [s^−1^]	0.039 ± 0.03	1.1 ± 0.15	0.436 ± 0.19	1.77 ± 0.39
	*X*_β_ [Å]	4.56 ± 0.37	4.29 ± 0.3	4.53 ± 0.51	3.43 ± 0.06
	*τ* [s]	25.6	0.91	2.29	0.56
*BPA*	*K*_on_ [M^−1^s^−1^]	5.7 × 10^4^	3.6 × 10^4^	1.7 × 10^4^	4.1 × 10^3^
	$K_{\mathrm{off}}^\prime$ [s^−1^]	1.3	0.35	0.82	–
	*τ*′ [s]	0.7	2.844	1.22	–
	*K*_D_ [mM] *=* $K_{\mathrm{off}}^\prime / K_{\mathrm{on}}$	0.023	0.009	0.048	–
	*(K*_off_/*K*_on_*)*	(0.0007)	(0.003)	(0.0254)	(0.43)

### DbpA/B has faster association kinetics with decorin than with laminin

It should be noted that the Bell–Evans model underlies the assumption of a single sharp and force-independent barrier transversed in the energy landscape along the force-driven bond-dissociation pathway. Thereby, *K*_off_ and *X*_β_, derived from fitting the DFS data, characterize the height and the width of the energy barrier, respectively. Kinetic off-rate constants determined from ensemble average methods such as SPR and QCM^26,27^, however, may deviate from DFS values, if a different energy well is prominent when no force is applied.

To investigate the binding dynamics with a different approach, we varied the time in which Dbps/ECM bonds may form during force-distance cycles (denoted as dwell time) and monitored the binding probability. Arising from the molecular flexibility provided by the PEG linker, bond formation may occur within a range of about 10 nm (length of the coiled linkers plus attached molecules) above the surface and during tip-surface contact (about 25 nm, see the linear slope in the force curves of Fig. 3b) for both the approaching and retracting part. Thus, the dwell time *t* is given by the overall sum of these lengths divided by the vertical scanning speed of the tip. Initially, the binding probability increased with the dwell time for all Dbps/ECM combinations (Fig. 3e, f). From this well-known behavior, the kinetic on-rate constant *K*_on_ was retrieved, by approximating with pseudo first-order kinetics according to ${\mathrm{d}}P(t)/{\mathrm{d}}t = K_{\mathrm{on}} \cdot C_{\mathrm{eff}}(1 - P(t))$ (eq. 2)^27,28^. The effective concentration *C*_eff_ is the number of binding partners. i.e., effectively one Dbps molecule on the tip, within the effective volume *V*_eff_ accessible for free equilibrium interaction. *V*_eff_ can be described as a half-sphere with radius *r*_eff_, with the latter being the sum of the equilibrium crosslinker length (3 nm) and the size of the proteins involved in binding (7 nm). In contrast to our earlier work, where the binding probability went into saturation over time^27^, we here observed a quasi-exponential decrease after a local maximum had been reached (Fig. 3e, f). A similar behavior was also observed for the adhesion of P-selectin to P-selectin glycoprotein-1^29^, which was interpreted to bear force-dependent rate constants that are important for supporting physiological leukocyte rolling. The decay of the binding probability with time comes from successive dissociation of Dbps from ECM, before the force detection limit of our SMFS experiments (about 5 pN) in the retraction part of the force-distance cycles has been reached^29^. Taking the unforced dissociation rate, *K*_off_, during this time into account, the overall biomolecular Dbps/ECM reaction kinetics is given by the differential equation ${\mathrm{d}}P(t)/{\mathrm{d}}t = K_{\mathrm{on}} \cdot C_{\mathrm{eff}}\left( {1 - P\left( t \right)} \right) - K_{\mathrm{off}}P(t)$ (eq. 3)^29^.

As a result of the binding probability assay (BPA) (Fig. 3e, f), data fitting using the above equation yielded a kinetic on-rate (*K*_on_) of DbpA/decorin binding of 5.7 × 10^4^ M^−1^s^−1^. This value was slightly larger when compared to DbpB/decorin, but considerably exceeded the binding rates of Dbps to laminin (Table 1). The kinetic off-rates, $K_{\mathrm{off}}^\prime$, arising from the decaying part of the binding probability time course *P(t)* (Fig. 3e, f), were transformed into average bond lifetimes measured at zero-force, *τ*′, using $\tau ^\prime = 1/K_{\mathrm{off}}^\prime$ (eq. 4) (Table 1). For the DbpA/decorin bond, *τ*′ taken from this analysis (0.7 s) was dramatically shorter (~35×) than *τ* determined from DFS experiments. This indicates that the bond becomes greatly stabilized under force load. A similar effect, but much less pronounced, was observed for DbpA/laminin, whereas the behavior for DbpB/decorin (Table 1) was reversed. *K*_off_ for DbpB/laminin was very slow under equilibrium conditions, as *P(t)* did not show any decay within the measurement time (Fig. 3f). The macroscopic thermodynamic equilibrium dissociation constants *K*_D_ were measured using an extended microtiter plate assay method and Scatchard plot analysis (Supplementary Fig. 3). By comparing the *K*_D_ values carried out with BPA and microtiter plate assay at varying concentrations, we found that both techniques show similar affinity values in the µM range, and that DbpB/decorin shows a slightly higher affinity than DbpA/decorin. From our results it is quite evident that dissociation of the Dbps/ECM complexes in solution do not follow the same dissociation path as when a force is applied to the bonds. The considerably longer bond life time of DbpA/decorin in SMFS experiments clearly indicates that a much higher dissociation energy barrier is involved when the bond is force-loaded. Strikingly different is the behavior of the DbpB/laminin bond, which is governed by a much lower barrier under force than at equilibrium.

## Discussion

During its infectious cycle, *B. burgdorferi* migrates through different shear stress environments. After tick transmission, the spirochete interacts with the cells lining the vascular lumen (high shear stress), traverses through the vessel wall (low shear stress), and finally infects target tissues (variable/low shear stress). Thereafter, when a new naive tick starts feeding on an infected host, the spirochete migrates “tick-wards” through the ECM (variable/low shear stress). Whereas some of the molecular interactions that enable efficient dissemination in high shear stress areas have been described^30,31^, the effects of adhesins on spirochetal motility in low shear stress niches are unknown.

As shown by others^32^, spirochetes are able to adopt distinct motility states and transition between them in the ECM as they disseminate within the host. Our results reveal that a possible strategy how spirochetes coordinate the transition between these motility states, is by forming various bonds of different mechanical properties that are tuned by external forces. The increased stability of DbpA/decorin when force-loaded is tailored for spirochetes to withstand shear stress, thereby facilitating dissemination within vascular endothelia in blood vessels. Similar bond behavior has been reported to be involved in cellular adhesion, including BBK32 of *B. burgdoferi*^30,31^, selectin-mediated binding^29^, or ClfA–fibrinogen interaction^33^. In the milieu with absence of significant shear stress, these binding interactions appear to be responsible for the “wriggling” and “lunging” motility states that *Borrelia* establishes for instance during colonization of the mouse dermis^32^. On the other hand, some of the bonds (as the most striking DbpB/laminin) are better adapted to mediate long-term stationary adhesions in equilibrium and short-term binding under force. This allows the spirochetes to detach easily underflow or during their rotatory movement.

Although certain binding interactions might prevail at a certain motility state, the differing kinetics between the DbpA/B proteins and the various components of the ECM need to work together synchronously as a single strategy to enhance motility through the ECM. It is not probable that spirochetes could actively influence which ECM components they will bind to and therefore the overall net effect of all interactions has to be translocation-enhancing. Additionally, DbpA and DbpB are not known to be downregulated at any phase of mammalian infection, as opposed to other infection-associated borrelial surface proteins such OspC or VlsE^34^, therefore it is not probable that some binding interactions are suppressed at any infection stage.

High torque generated by the flagellar motor is the essential player in generating the force needed for spirochetes to migrate^35^. However, to be able to squeeze through dense, gel-like matrices with pore sizes much smaller than the diameter of their body, spirochetes need to form transiently stable interactions that facilitate pushing against the adjacent surface^32^. We show that DbpA/B are multifunctional proteins, which through binding either directly to ECM proteins or their associated glycosaminoglycans side chains^36^, are capable to propel and coordinate the movement within the ECM. The relatively weak forces between DbpA/B and ECM, when compared with higher binding forces of adhesins in nonmotile pathogenic bacteria^37–39^, suggest that the spirochetes maintain a very dynamic state in an infected host^30–32^. Together, these interactions help spirochetes to reach extraordinary high speeds^40^, which enable them to colonize a host and to avoid clearance by the immune system of a host more efficiently. As it is known that bacteria are able to sense and respond to physical stimuli to optimize their function and overall fitness^41^, an attractive target of future studies could be to find out whether mechanical stress, experienced by spirochetes for instance as they penetrate the tight gel matrices, leads to upregulation of proteins such as DbpA/B.

In summary, we showed that spirochetes, despite a limited number of adhesive molecules, are able to leverage a wide variety of adhesion strategies in order to streamline their dissemination through the host. This unique mode of motility enhancement in low shear stress environment exploiting transient binding of the spirochete to adjacent surfaces might be common for many pathogenic organisms that are able to effectively migrate through dense, gel-like matrices^42,43^.

## Methods

### Ethics statement

All tick and animal experiments were approved by the BC ASCR animal ethical committee (Animal protection laws of the Czech Republic No. 246/1992 Sb., Ethics approval No. 79/2013). All experiments were performed in accordance with relevant guidelines and regulations.

### Bacterial strains and culture conditions

*E. coli* strains DH5α (NEB), NEB Express Iq (NEB) and M15 [pREP4] (Qiagen) were grown with aeration in Lysogeny broth at 37 °C under antibiotic selection with ampicillin (100 μg/mL) or kanamycin (50 μg/mL), where appropriate.

Recombinant *B. burgdorferi* B313 strains (B313/DbpAB, B313/BBK32, B313/RevA) expressing DbpA and DbpB, BBK32, and RevA of *B. afzelii* A91 were used in this study. B313/DbpAB together with the control strain containing empty shuttle vector pBSV2 (B313/pBSV2) were prepared elsewhere^6^. These isolates together with *B. burgdorferi* B31, *B. burgdorferi* B313, and *B. afzelii* A91 were cultured in Barbour–Stoenner–Kelly (BSK)-H medium (Sigma-Aldrich) at 34 °C.

### Generation of recombinant DbpA, DbpB, RevA, and BBK32 proteins

To generate recombinant histidine-tagged RevA and BBK32 proteins, the *revA* and *bbk32* open reading frames lacking the putative signal sequences from *B. afzelii* A91 were amplified using the primers described in Supplementary Table 1. Amplified fragments were engineered to encode a BamHI site at the 5′ end and a HindIII site at the 3′ end. Amplified DNA fragments were inserted into TA cloning vector pGEM-T Easy (Promega). The resulting plasmids were then digested with BamHI and HindIII to release the genes, which were then inserted into the pQE30 (Qiagen) predigested with BamHI and HindIII. The resulting plasmids were transformed into *E. coli* NEB Express Iq (NEB) and the plasmid inserts were sequenced. The construction of recombinant strains *E. coli* M15 expressing DbpA and DbpB from *B. afzelii* A91 is described elsewhere^44^.

Large cultures were induced with the use of isopropyl *β*-D-1-thiogalactopyranoside (final concentration, 0.5 mM) and lysed by sonication. Recombinant soluble proteins were harvested and purified under native conditions using Protino Ni-NTA Agarose (Macherey-Nagel). Bound proteins were eluted with 250 mM imidazole in TRIS buffer (50 mM, pH 8, 300 mM NaCl). The proteins were washed, desalted, and concentrated in Amicon Ultra-15 3000 MWCO (Merck Millipore). Purity was checked using SDS-PAGE and protein concentrations were measured using Bradford assay.

### Immunization of rabbit with DbpA, DbpB, RevA, and BBK32

Female New Zealand white rabbits (Velaz, Czech Republic) were immunized by injection subcutaneously with 100 μg recombinant DbpA or DbpB from *B. afzelii* A91 in TRIS buffer (1:1) with complete Freund’s adjuvant (Sigma-Aldrich). The rabbit received two boosts of 100 μg recombinant DbpA or DbpB in TRIS buffer (1:1) with incomplete Freund’s adjuvant (Sigma-Aldrich) at 14-day intervals. One week after the final boost, the rabbit was bled to obtain serum. The serum was tested qualitatively by Western blotting to determine the specificity of the antiserum for DbpA and DbpB against recombinant DbpA protein and *B. burgdorferi* lysates. Briefly, proteins were separated by SDS-polyacrylamide gel electrophoresis and transferred to nitrocellulose. Membranes were blocked for 2 h with 5% BSA in TRIS-buffered saline with 0.05% Tween 20 (TBS-T). Membranes were washed with TBS-T and incubated for 2 h at room temperature (RT) with DbpA/DbpB antisera diluted 1:200 in blocking buffer. After washing with TBS-T, membranes were incubated for 1 hr at RT with goat anti-rabbit immunoglobulin G conjugated with horseradish peroxidase (HRP) (Vector Laboratories) diluted 1:10,000 in blocking buffer. After a final series of washes with TBS-T, bound antibodies were detected by using Pierce ECL western blotting chemiluminescence substrate (Thermo Scientific).

### Generation and characterization of B313/BBK32 and B313/RevA

To generate the shuttle vectors expressing BBK32 and RevA proteins, the respective genes with upstream regions were first PCR amplified with the addition of a HindIII site and a BamHI site at the 5′ and 3′ ends, respectively, using the primers listed in Supplementary Table 1. Amplified DNA fragments were inserted into TA cloning vector pGEM-T Easy (Promega). The resulting plasmids were then digested with HindIII and BamHI to release the genes, which were then inserted into the shuttle vector pBSV2K predigested with HindIII and BamHI.

*B. burgdorferi* B313 was transformed by electroporation with 40 µg of each of the shuttle vectors and cultured in BSK-H medium at 34 °C for 24 h. The transformants were selected in BSK1.5× semisolid medium containing 1.7% agarose and 50 μg/mL kanamycin at 34 °C for 2 weeks. *B. burgdorferi* transformants were confirmed by colony PCR using kanamycin specific primers. The presence of RevA/BBK32 was confirmed by Western blot assay using sera from a rabbit immunized with recombinant RevA/BBK32 from *B. afzelii* A91.

Surface localization of the adhesins was performed with proteinase K assay similarly as in the previous studies^6,45^. Proteinase K was added to the cell suspension at a final concentration of 100 μg/mL, incubated at RT for 3 min, and washed twice with PBS before preparing the samples for Western blot analyzes. Anti-flagellin antibody (Rockland) (1:5000) with anti-rabbit HRP as a secondary antibody (1:10,000) were used. Protein Marker VI (10–245) prestained (AppliChem) was used for all Western blots.

### Plate growth and swarm motility assays

For growth assay, BSK 1.5× medium for semisolid plating was prepared as described by Samuels^46^ and 100 spirochetes were plated by mixing with BSK 1.5× in a petri dish. Plates were incubated at 34 °C and for 3 weeks in a microaerophilic chamber. Colony images were obtained in a Bio-Rad ChemiDoc Imaging System and colony diameters were measured using ImageJ. Thirty colony diameters were measured for each strain. One-way ANOVA test was used to compare the colony diameters.

Spirochete cell motility was determined by swarm plate assays. Swarm plates were prepared by mixing 50 mL BSK 1.5× medium, 144 mL Dulbecco’s Phosphate Buffered Saline (PBS), and 60 mL 1.7% agarose. Approximately 1 × 10^6^ cells in a volume of 5 μL were spotted onto plates. Swarm plates were incubated at 34 °C and for 3 weeks in a microaerophilic chamber. Statistical tests were performed as stated above for growth assay.

### In vitro tick feeding assay

Pathogen-free adult females of *I. ricinus* reared in our in-house facility were used for the experiments. The feeding units for membrane feeding of ticks were prepared according to the procedure developed by Kröber and Guerin^47^. Rabbit blood was collected in the animal house facility of the Institute of Parasitology, Biology Center ASCR and manually defibrinated. For feeding, 10 ticks were placed in the feeding unit. To initiate tick feeding, blood was added into the feeding unit and regularly exchanged at intervals of 8 h. After 24 h, unattached females were removed. To study the motility of *Borrelia*, 100 μL of ECM Gel from Engelbreth-Holm-Swarm murine sarcoma (Sigma-Aldrich, E6909) and 600 μL of *Borrelia* cultures (5 × 10^6^ cells) were first mixed on ice and then thoroughly mixed with 500 μL of 2% low melting point agarose (SeaPlaque^TM^, Lonza, 50100) heated to 60 °C and placed into the six-well cell culture plate (Corning Costar). Once solidified, 3.5 mL of sterile-filtered rabbit serum (Sigma, R4505) was pipetted onto the gel-embedded bacteria. The feeding units with ticks were submerged into the serum and placed in a water bath heated to 37 °C. Samples of rabbit serum were collected in duplicates (2 × 200 μL) at 1-hr interval, in total 4 h. For inhibition experiments, the spirochetes were preincubated with decorin (10 μg) from bovine articular cartilage (Sigma-Aldrich) at 37 °C for 30 min and the feeding assay was done as described above. The unpaired Student *t* test was used to test the statistical significance.

### DNA isolation and real-time quantitative PCR

DNA isolation from rabbit sera was performed using the Nucleospin Blood kit as per manufacturer’s instructions (Macherey-Nagel). DNA extracts were examined for *B. burgdorferi* spirochetes by using a real-time quantitative PCR (qPCR) assay with Fast Start Universal SYBR Green Master Kit (Roche) and primers specific for a section of the 16S rRNA gene^48^, listed in Supplementary Table 1. To assess spirochete density per sample, a standard curve was generated in log increments (10–10^6^). The numbers of borrelial genomic copies were calculated by comparing the threshold cycle (*C*_*T*_) values with the values for serial dilutions of known amounts of *B. burgdorferi* genomic DNA, which were used as standards. Samples were analyzed using a LightCycler 480 (Roche). The PCR conditions were as follows: 95 °C for 5 min, followed by 50 cycles of 95 °C for 15 s, 60 °C for 15 s, and 72 °C for 15 s.

### Adhesion of recombinant DbpA and DbpB to ECM gel and ECM components

Specific interactions between borrelial adhesins and ECM gel components were detected using far western blotting. Briefly, 2 μL of ECM gel was separated by SDS-polyacrylamide gel electrophoresis and after blotting and blocking with 2.5% BSA, recombinant DbpA and DbpB (50 μg) were added, followed by incubation with anti-HisTag-HRP labeled antibody (R&D Systems).

The Nunc MaxiSorp ELISA plates (Thermo Scientific) were coated with 1 μg laminin from human fibroblasts (Sigma-Aldrich), recombinant human collagen Type I (Sigma-Aldrich), human fibronectin (R&D Systems), or with decorin from bovine articular cartilage (Sigma-Aldrich) in 100 μL/well coating buffer (100 mM bicarbonate/carbonate buffer [pH 9.6]) overnight at 4 °C, and blocked with 200 μL of 2.5% BSA in TRIS buffer for 1 h at RT. Subsequently, the respective DbpA (0.5 μg/well in 100 μL TRIS buffer) was added, incubated for 1 h at RT, and washed (wash buffer; TRIS plus 0.05% Tween-20), followed by incubation with anti-HisTag-HRP (1:10,000) labeled antibody in 50 μL of blocking buffer for 30 min at RT and five washes. Finally, tetramethylbenzidine substrate (Sigma-Aldrich) was added. The reaction was stopped with 1 M sulfuric acid and the absorbance at 450 nm was determined using the Infinite 200 M Pro microplate reader (Tecan). For the extended microtiter plate assay method we varied the concentration of the ligand and fitted the data using a Scatchard plot by applying the following equation to calculate the dissociation constant *K*_D_, *B*/*F* = 1/ *K*_D_(*B*_max_ – *B*), where *B* equals the bound molecules and *F* the free ligand concentration^49^.

### Conjugation of borrelial surface proteins through histidine residues of His-tagged protein

A maleimide-Poly(ethylene glycol) (PEG) linker was attached to a 3-aminopropyltriethoxysilane (APTES)-coated AFM cantilever by incubating the cantilevers for 2 h in 500 µL of chloroform containing 1 mg of maleimide-PEG-N-hydroxysuccinimide (NHS) (Polypure) and 30 µl of triethylamine. After three times washing with chloroform and drying with nitrogen gas, the cantilevers were immersed for 2 h in a mixture of 100 µL of 2 mM thiol-trisNTA, 2 µL of 100 mM EDTA (pH 7.5), 5 µL of 1 M HEPES (pH 7.5), 2 µl of 100 mM tris(carboxyethyl)phosphine (TCEP) hydrochloride, and 2.5 µL of 1 M HEPES (pH 9.6) buffer, and subsequently washed with HEPES buffer. Thereafter, the cantilevers were incubated for 4 h in a mixture of 4 µL of 5 mM NiCl_2_ and 100 µL of 0.2 µM His-tagged borrelial surface proteins (DbpA, DbpB). After washing three times with HEPES-buffer saline (HBS), the cantilevers were store in HBS at 4 °C^21^.

### Conjugation of extracellular matrix (decorin, laminin) through lysine residues to the substrate

1 × 1 cm^2^ silicon nitride substrates were coated with APTES, before a heterobifunctional acetal-PEG linker was attached via its NHS ester group for coupling of decorin from bovine articular cartilage (Sigma-Aldrich) or laminin from human fibroblasts (Sigma-Aldrich) via one of the lysine residues. The bond was fixed by reduction with NaCNBH_3_. The overall procedure was done as described before^50^. Substrates were washed and stored in PBS at 4 °C before measurements.

### Single-molecule force spectroscopy (SMFS)

SMFS measurements were performed at RT using borrelial surface protein conjugated tips with 0.01 N/m nominal spring constants (MSCT, Bruker). The deflection sensitivity was calculated from the slope of the force-distance curves recorded on a bare silicon substrate. Force-distance curves were acquired by recording at least 1000 curves with vertical sweep times between 0.5 and 10 s and at a z-range of typically 500 nm, resulting in loading rate from 100 to 10,000 pN/s, using a commercial AFM (Keysight Technologies, USA). The relationship between experimentally measured unbinding forces and the interaction potential is described by kinetic models^24,25^. Blocking of a specific interaction between borrelial surface protein and extracellular matrix was done by injecting borrelial surface proteins into the bath solution, which resulted in a de-activated ECM on the surface. All SMFS experiments were repeated on at least five different preparations of functionalized AFM cantilevers and sample surfaces.

### Statistics and reproducibility

The details about experimental design and statistics used in different data analyzes performed in this study are given in the respective sections of results, figure legends, and methods. All AFM experiments were performed at least three times independently. Data are presented as mean ± standard error of the mean. The number of experiments, the name of the statistical test, and exact *p* values are provided in each figure legend. Comparisons between groups were determined using Student’s *t* test or ANOVA. Differences were considered significant at *P < 0.05; **P < 0.01; ***P < 0.001.

### Reporting summary

Further information on research design is available in the Nature Research Reporting Summary linked to this article.

## Supplementary information

Supplementary Information

Description of Additional Supplementary Files

Suplementary Data 1

Reporting Summary

## Data Availability

The datasets generated or analyzed during the current study are available from the corresponding author on reasonable request. Source data underlying plots shown in figures are provided in Supplementary Data 1. Uncropped scans of Western blots are shown in Supplementary Fig. 4.
