# Supplementary material for: Nanomechanical mechanisms of Lyme disease spirochete motility enhancement in extracellular matrix
Source: Commun Biol. 2021 Mar 1;4:268. doi: 10.1038/s42003-021-01783-1 (PMC7921401; doi:10.1038/s42003-021-01783-1)
Supplement: Supplementary file 1 — Supplementary Information [file 42003_2021_1783_MOESM1_ESM.pdf]

Supplementary information

Nanomechanical mechanisms of Lyme disease spirochete motility enhancement in extracellular matrix

Martin Strnad[1,2,*], Yoo Jin Oh[3,*], Marie Vancová[1,2], Lisa Hain[3], Jemiina Salo[4], Libor Grubhoffer[1,2], Jana Nebesářová[1,2], Jukka Hytönen[5], Peter Hinterdorfer[3] and Ryan O.M. Rego[1,2]

[1]*Biology Centre ASCR, v.v.i., Branisovska 31, 370 05 Ceske Budejovice, Czech Republic*

[2]*Faculty of Science, University of South Bohemia, Branisovska 31, 370 05 Ceske Budejovice, Czech Republic*

[3]*Institute of Biophysics, Johannes Kepler University Linz, Gruberstrasse 40, A-4020, Linz, Austria*

[4]*Institute of Biomedicine, University of Turku, FI-20014, Turku, Finland*

[5]*Laboratory Division, Clinical Microbiology, Turku University Hospital, 20520, Turku, Finland*

These authors contributed equally: M. Strnad and Y. J. Oh

Corresponding authors: M. Strnad and Y. J. Oh

Email: martin.strnad.cze@gmail.com; yoo_jin.oh@jku.at

Supplementary Table 1: Primers used in this study.

Primer/Vector	Sequence*	Amplified DNA fragment
RevA/pQE30 F	cGGATCCaaattatatgtaaaagaaaaagaag	*revA*$_{A91}$
RevA/pQE30 R	cAAGCTTtcaattagtaccttcttcta	
BBK32/pQE30 F	cGGATCCttattcataagagatgaaataaaaga	*bbk32*$_{A91}$
BBK32/pQE30 R	cAAGCTTttagtaccaaacaccattc	
RevA/pBSV2K F	cAAGCTTgattttaattgaaagatttatatttttaga	*revA*$_{A91}$*+upstream region*
RevA/pBSV2K R	aGGATCCtcaattagtaccttcttctagaa	
BBK32/pBSV2K F	cAAGCTTttggctgttgtaattaatgctt	*bbk32*$_{A91}$*+upstream region*
BBK32/pBSV2K R	aGGATCCttagtaccaaacaccattctta	
Kan F	atgagccatattcaacgggaa	Kanamycin
Kan R	ttagaaaaactcatcgagcat	
16S rRNA F	gctgtaaacgatgcacacttggt	16S rRNA
16S rRNA R	ggcggcacacttaacacgttag	

* The restriction sites used are underlined and in capital letters.

Supplementary Figure 1: Recombinant DbpA and DbpB mediate highest binding to decorin and laminin

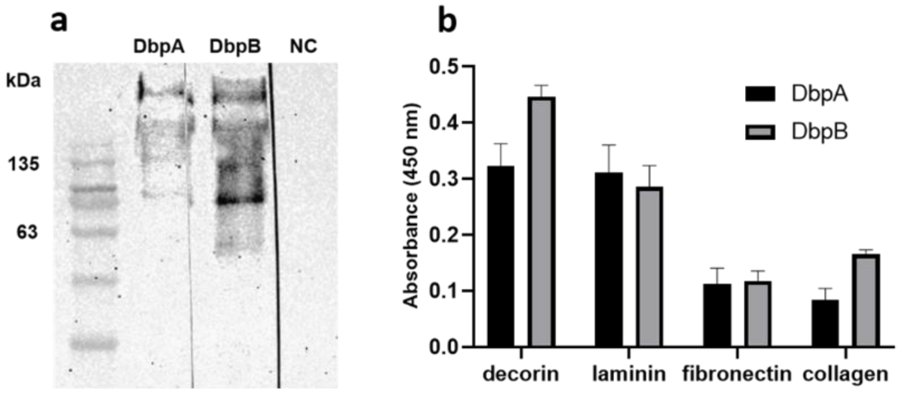

a, DbpA and DbpB mediate binding to multiple ECM gel components, as shown by far-western blot analysis (NC; negative control). **b,** The highest binding was measured for decorin and laminin using microtiter plate assay. Bound DbpA and DbpB was detected by specific anti-HisTag antibodies. Results are expressed as arithmetic mean of OD450 values, subtracted with control BSA values. Error bars, standard deviation of three experiments.

Supplementary Figure 2: A plot of dissociation forces vs. loading rates for each Dbps/ECM analog

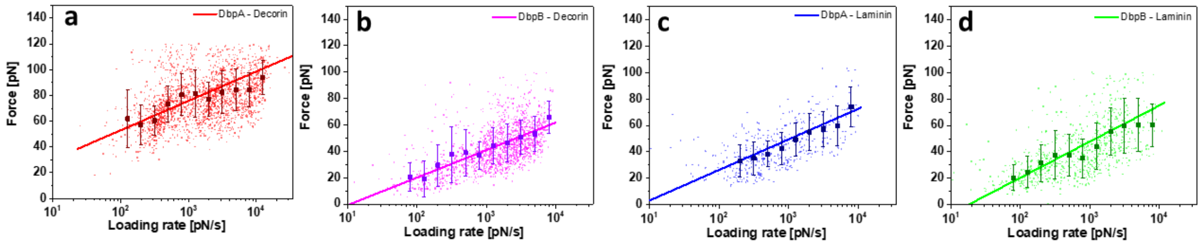

Individual dissociation forces *vs.* loading rates for (**a**) DbpA/decorin, (**b**) DbpB/decorin, (**c**) DbpA/laminin, and (**d**) DbpB/laminin. 5 different loading rates with 1000 force curves each were recorded for each plot and tested with at least 3 different cantilevers and surface preparations. Data scatterings reflect the stochastic nature of the dissociation process, rather than the experimental uncertainty. Data were fitted according to the equation of Bell and Evans[24,25], using a maximum likelihood approach and the Bayesian information criterion to select the best fitting. The force loading rate r_i of every individual force curve was calculated by multiplying the pulling velocity with the effective spring constant, calculated from 2 springs in parallel, i.e. the spring constant of the cantilever and the molecules at the point of bond dissociation[27]. Each measured unbinding force was plotted as a

function of its loading rate. The data variance reflects the stochastic nature of the unbinding process and not the measurement error. Averages of measured unbinding forces and their standard deviations are indicated.

Supplementary Figure 3: Determination of the dissociation constants of Dbps/ECM based on microtiter plate assay

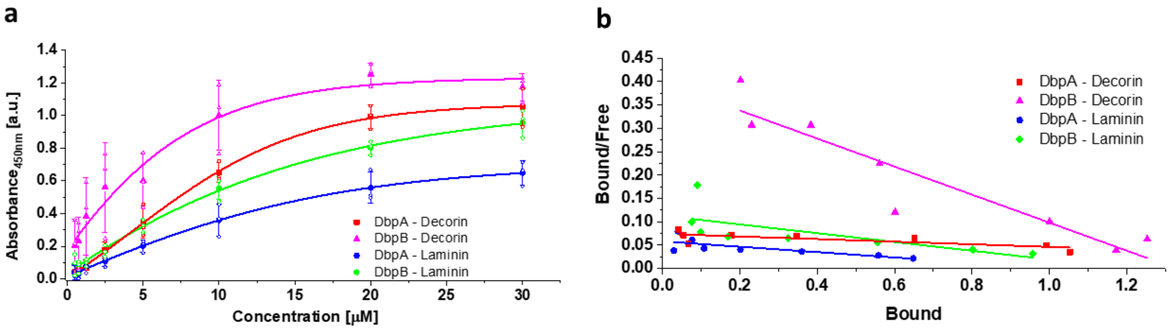

a, Extended microtiter plate assay curves measured between Dbps/ECM at different concentrations, and (**b**) Scatchard plot ($Y_{(Bound/Free)}$=1/ K_D(1- $X_{(Bound)}$)) used for determining the equilibrium dissociation constant K_D for each Dbps/ECM complex. Bound was determined from the ELISA curve and Free represents the ligand concentration. The calculated values were: DbpA/decorin: 35.99 µM, DbpB/decorin: 3.34 µM, DbpA/laminin: 17.37 µM, DbpB/laminin: 10.65 µM.

Supplementary Figure 4: Complete western blots illustrated in Fig. 1a. All lanes and antibodies used for staining are shown.

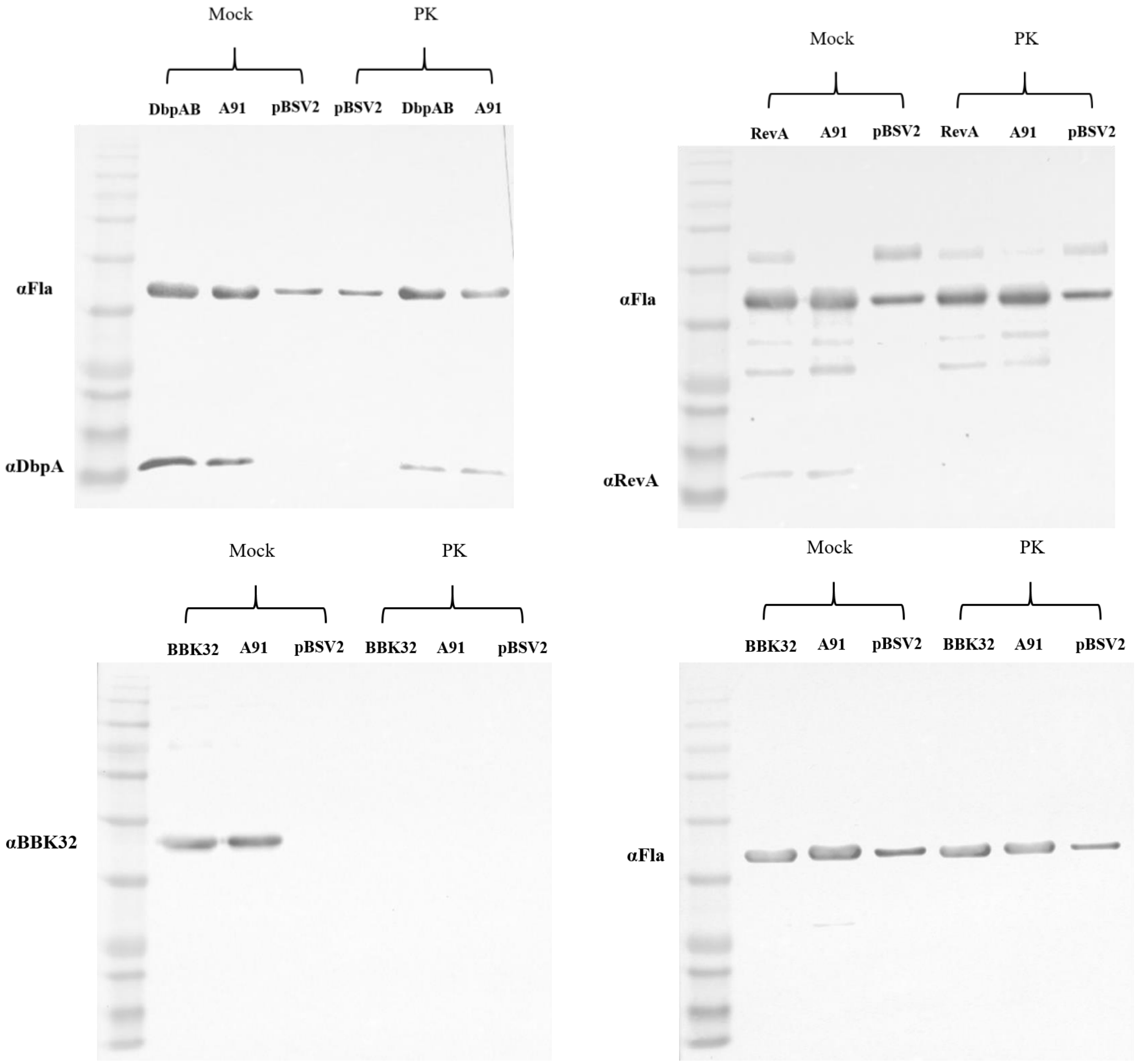

Wild type *B. afzelii* A91 was used as positive control, *B. burgdorferi* B313/pBSV2 as negative control.
