# Supplementary material for: Nanomechanical mechanisms of Lyme disease spirochete motility enhancement in extracellular matrix
Source: Commun Biol. 2021 Mar 1;4:268. doi: 10.1038/s42003-021-01783-1 (PMC7921401; doi:10.1038/s42003-021-01783-1)
Supplement: Supplementary file 2 — Description of Additional Supplementary Files [file 42003_2021_1783_MOESM2_ESM.pdf]

Description of Additional Supplementary Files

File name: Supplementary Data 1

Description:

Fig. 1b - The effect of DbpAB, RevA, and BBK32 on undirected movement of spirochetes was studied using a growth assay. Borrelia carrying empty shuttle vector pBSV2 were used as the negative control. A diameter of 30 colonies was measured.

Fig. 1c - The effect of DbpAB, RevA, and BBK32 on undirected movement of spirochetes was studied using swarm motility assay. Borrelia carrying empty shuttle vector pBSV2 were used as the negative control. A diameter of 12 colonies was measured.

Fig. 2d - The effect of DbpAB, RevA, and BBK32 on directed movement of spirochetes was studied using a tick feeding assay. The number of spirochetes that reached rabbit serum was monitored over time using qPCR. The values are given as percentage of the total number of spirochetes seeded to the ECM gel. Samples of rabbit serum were collected at 1-hr interval, in total 4 hrs. Borrelia carrying empty shuttle vector pBSV2 were used as the negative control. B. afzelii A91 was used as the positive control.

Fig. 2e-g - The effect of decorin on directed movement of DbpAB/B313 was studied using a tick feeding assay. The number of spirochetes that reached rabbit serum was monitored over time using qPCR. The values are given as percentage of the total number of spirochetes seeded to the ECM gel. Samples of rabbit serum were collected at 1-hr interval, in total 4 hrs. Borrelia carrying empty shuttle vector pBSV2 were used as the negative control. B. afzelii A91 was used as the positive control.

Fig. 3b - Force-distance curves obtained by SMFS

Fig. 3c - Probability density fucntions vs. forces

Fig. 3d - Dissociation force vs. force loading rate

Fig. 3e-f - Binding probability vs. dwell time
