# Supplementary material for: Nanomechanical mechanisms of Lyme disease spirochete motility enhancement in extracellular matrix
Source: Commun Biol. 2021 Mar 1;4:268. doi: 10.1038/s42003-021-01783-1 (PMC7921401; doi:10.1038/s42003-021-01783-1)
Supplement: Supplementary file 4 — Reporting Summary [file 42003_2021_1783_MOESM4_ESM.pdf]

nature research

Corresponding author(s): Martin Strnad, MSc., Dr. Yoo Jin Oh

Last updated by author(s): Jan 18, 2021

Reporting Summary

Nature Research wishes to improve the reproducibility of the work that we publish. This form provides structure for consistency and transparency in reporting. For further information on Nature Research policies, see our Editorial Policies and the Editorial Policy Checklist.

Statistics

For all statistical analyses, confirm that the following items are present in the figure legend, table legend, main text, or Methods section.

n/a	Confirmed	
☐	☒	The exact sample size (*n*) for each experimental group/condition, given as a discrete number and unit of measurement
☐	☒	A statement on whether measurements were taken from distinct samples or whether the same sample was measured repeatedly
☐	☒	The statistical test(s) used AND whether they are one- or two-sided<br>*Only common tests should be described solely by name; describe more complex techniques in the Methods section.*
☒	☐	A description of all covariates tested
☐	☒	A description of any assumptions or corrections, such as tests of normality and adjustment for multiple comparisons
☐	☒	A full description of the statistical parameters including central tendency (e.g. means) or other basic estimates (e.g. regression coefficient) AND variation (e.g. standard deviation) or associated estimates of uncertainty (e.g. confidence intervals)
☐	☒	For null hypothesis testing, the test statistic (e.g. F, t, r) with confidence intervals, effect sizes, degrees of freedom and P value noted<br>*Give P values as exact values whenever suitable.*
☒	☐	For Bayesian analysis, information on the choice of priors and Markov chain Monte Carlo settings
☒	☐	For hierarchical and complex designs, identification of the appropriate level for tests and full reporting of outcomes
☒	☐	Estimates of effect sizes (e.g. Cohen's d, Pearson's r), indicating how they were calculated

Our web collection on statistics for biologists contains articles on many of the points above.

Software and code

Policy information about availability of computer code

Data collection	Was done manually
Data analysis	Data analysis was performed using an in-house written algorithm programmed in Matlab.

For manuscripts utilizing custom algorithms or software that are central to the research but not yet described in published literature, software must be made available to editors and reviewers. We strongly encourage code deposition in a community repository (e.g. GitHub). See the Nature Research guidelines for submitting code & software for further information.

Data

Policy information about availability of data

All manuscripts must include a data availability statement. This statement should provide the following information, where applicable:

- Accession codes, unique identifiers, or web links for publicly available datasets
- A list of figures that have associated raw data
- A description of any restrictions on data availability

The authors declare that the data supporting the findings of this study are available within the paper and its supplementary files or available from the corresponding author upon reasonable request.

Field-specific reporting

Please select the one below that is the best fit for your research. If you are not sure, read the appropriate sections before making your selection.

- [x] Life sciences
- [ ] Behavioural & social sciences
- [ ] Ecological, evolutionary & environmental sciences

For a reference copy of the document with all sections, see nature.com/documents/nr-reporting-summary-flat.pdf

Life sciences study design

All studies must disclose on these points even when the disclosure is negative.

Sample size	Sample sizes was based on the minimum required to calculate obtained results statistically.
Data exclusions	No data was excluded from the study
Replication	30 colony diameters were measured for each mutant for colony formation and at least 12 each for swarm motility assay. Minimum of 3 to 4 replicates were carried out for the in vitro tick feeding assay. the All SMFS experiments were repeated on at least five different preparations of functionalized AFM cantilevers and sample surfaces.
Randomization	The study was to look at differences in binding properties of the particular proteins studies in this work and therefore there was no current need for randomized experiments
Blinding	Blinding is not relevant as we were looking to understand the binding properties of the proteins studies within this work .Also the physical properties that are attributed based on experimental evidence was clearly based on the particular protein being expressed and investigated. The questions being asked were direct and hence no reason to have a blinded study.

Reporting for specific materials, systems and methods

We require information from authors about some types of materials, experimental systems and methods used in many studies. Here, indicate whether each material, system or method listed is relevant to your study. If you are not sure if a list item applies to your research, read the appropriate section before selecting a response.

Materials & experimental systems

n/a	Involved in the study	
☐	☒	Antibodies
☒	☐	Eukaryotic cell lines
☒	☐	Palaeontology and archaeology
☐	☒	Animals and other organisms
☒	☐	Human research participants
☒	☐	Clinical data
☒	☐	Dual use research of concern

Methods

n/a	Involved in the study	
☒	☐	ChIP-seq
☒	☐	Flow cytometry
☒	☐	MRI-based neuroimaging

Antibodies

Antibodies used	The only commercial antibody used was R&D Systems - His Tag Horseradish Peroxidase-conjugated Antibody MAB050H, Lot No: EGE1217121 and Rockland anti-flagellin antibody (200-401-C14S). All other antibodies used in this study were generated in house , raised in rabbits.
Validation	All relevant work related with validation of this antibody is provided at the url https://www.rndsystems.com/products/his-tag-horseradish-peroxidase-conjugated-antibody-ad1110_mab050h

Animals and other organisms

Policy information about studies involving animals; ARRIVE guidelines recommended for reporting animal research

Laboratory animals	New Zealand white rabbit were purchased from Velaz, Czech Republic. 3-4 months old females were used for antibody production
Wild animals	Study did not involve wild animals.
Field-collected samples	Ixodes ricinus adult females were obtained from the breeding facility of the Institute of Parasitology, Biology Centre, Czech Academy of Sciences. Ticks were maintained in wet chambers with a humidity of about 95%, temperature of 24°C, and day/night period set to 15/9h.

Ethics oversight	All tick and animal experiments were approved by the BC ASCR animal ethical committee (Animal protection laws of the Czech Republic No. 246/1992 Sb., Ethics approval No. 79/2013). All experiments were performed in accordance with relevant guidelines and regulations.

Note that full information on the approval of the study protocol must also be provided in the manuscript.
